# Killing Two Cells with One Stone: Pharmacologic BCL-2 Family Targeting for Cancer Cell Death and Immune Modulation

**DOI:** 10.3389/fped.2016.00135

**Published:** 2016-12-21

**Authors:** Lindsey M. Ludwig, Michele L. Nassin, Abbas Hadji, James L. LaBelle

**Affiliations:** ^1^Section of Hematology, Oncology, Stem Cell Transplantation, Department of Pediatrics, University of Chicago, Comer Children’s Hospital, Chicago, IL, USA; ^2^Committee on Cancer Biology, University of Chicago, Chicago, IL, USA

**Keywords:** BCL-2, lymphocytes, BH3 mimetic, apoptosis, cell death, immune system, small molecules, immunotherapy

## Abstract

A crucial component of regulating organismal homeostasis is maintaining proper cell number and eliminating damaged or potentially malignant cells. Apoptosis, or programed cell death, is the mechanism responsible for this equilibrium. The intrinsic apoptotic pathway is also especially important in the development and maintenance of the immune system. Apoptosis is essential for proper positive and negative selection during B- and T-cell development and for efficient contraction of expanded lymphocytes following an immune response. Tight regulation of the apoptotic pathway is critical, as excessive cell death can lead to immunodeficiency while apoptotic resistance can lead to aberrant lymphoproliferation and autoimmune disease. Dysregulation of cell death is implicated in a wide range of hematological malignancies, and targeting various components of the apoptotic machinery in these cases is an attractive chemotherapeutic strategy. A wide array of compounds has been developed with the purpose of reactivating the intrinsic apoptotic pathway. These compounds, termed BH3 mimetics are garnering considerable attention as they gain greater clinical oncologic significance. As their use expands, it will be imperative to understand the effects these compounds have on immune homeostasis. Uncovering their potential immunomodulatory activity may allow for administration of BH3 mimetics for direct tumor cell killing as well as novel therapies for a wide range of immune-based directives. This review will summarize the major proteins involved in the intrinsic apoptotic pathway and define their roles in normal immune development and disease. Clinical and preclinical BH3 mimetics are described within the context of what is currently known about their ability to affect immune function. Prospects for future antitumor immune amplification and immune modulation are then proposed.

## The Apoptotic Pathway and BCL-2 Family Proteins

**Figure 1:**
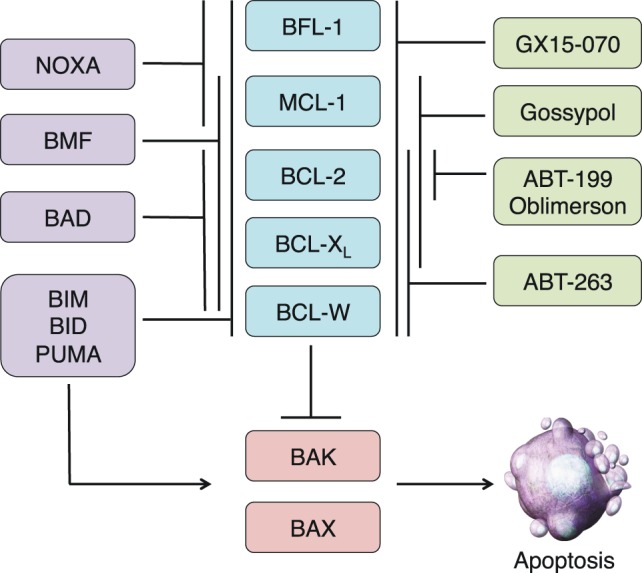
**Overview of the BCL-2 family and BH3 mimetics in clinical trials**. The BCL-2 family is divided into three subgroups: the multidomain antiapoptotics (blue), the multidomain proapoptotics (red), and the BH3-only proteins (purple). The antiapoptotic proteins sequester the proapoptotic proteins BAX and BAK. In times of cellular stress, BH3-only proteins can either bind to the antiapoptotic proteins and release the proapoptotics from their sequestration or directly bind and activate BAX and BAK. Once activated, BAX and BAK oligomerize and induce mitochondrial outer membrane permeabilization, leading to the release of other proapoptotic factors and eventual cellular destruction. The actions of the BH3-only proteins can be imitated by BH3 mimetics, some of which have reached clinical trials (green). Like the BH3-only proteins, these compounds have varying specificities for the antiapoptotic proteins.

The apoptotic cascade can be divided into two main pathways, both of which culminate in the activation of effector caspases that cleave essential substrates and in turn mediate the ultimate destruction of the cell (1, 2). The extrinsic pathway is initiated through external signals propagated *via* death receptors on the cell surface such as FAS (CD95) or other members of the tumor necrosis factor receptor (TNFR) family. Ligand-induced receptor trimerization initiates cellular demise through adaptor protein association and initiator caspase-8 activation (3, 4). In contrast, the intrinsic pathway is activated in response to a variety of internal cellular stresses and is mediated primarily by the BCL-2 family of proteins. BCL-2 was first discovered as a part of a chromosomal translocation in B-cell lymphoma and was the first known oncogene to inhibit cell death as opposed to actively promoting proliferation (5–7). The BCL-2 proteins share one to four highly conserved regions in both sequence and structure, termed BCL-2 homology (BH) domains. Based on these domains, and in conjunction with their activity profile, the BCL-2 family is divided into three functional subgroups: the multidomain antiapoptotics (BCL-2, BCL-X_L_, BCL-W, MCL-1, BFL-1), the multidomain proapoptotics (BAK, BAX, BOK), and the BH3-only proteins (BIM, BID, BAD, NOXA, PUMA, BMF, BIK, HRK) (Figure 1). The BH3-only proteins, named so because they share only the third BH domain with the other BCL-2 family proteins, act as cellular sentinels that in times of stress bind discrete multidomain BCL-2 proteins and initiate the apoptotic cascade (8). This process can occur through two known mechanisms. BH3-only proteins can bind antiapoptotic BCL-2 members causing release of sequestered BAX and BAK (9). These are *indirectly* activating BH3-only proteins (e.g., BAD and NOXA). In addition, other BH3-only proteins, such as BIM, BID, and PUMA, can not only bind antiapoptotics but are also able to *directly* bind and activate BAK and BAX oligomerization (10). Once oligomerized, BAK and BAX form pores in the outer mitochondrial membrane causing mitochondrial outer membrane permeabilization (MOMP), which leads to the release of cytochrome *c* and other proapoptotic factors such as SMAC/DIABLO from the inner mitochondrial membrane space (11, 12). Cytochrome *c* associates with APAF and caspase-9 to form the apoptosome, which initiates the cleavage of effector caspases 3 and 7 leading to eventual cellular destruction (13). The contact interfaces between antiapoptotic and BH3-only proteins have been elucidated through crystal structure analyses. This has led to increasing interest and ability to design drugs that recapitulate these interactions in an effort to overcome apoptotic resistance. While these efforts have mainly focused on inducing cell death in the context of cancer therapy, there is potential to use these compounds as immunomodulators based upon the differential BCL-2 family member dependencies of immune cells (14).

## The Role and Potential Targeting of BCL-2 Proteins in the Immune System

### Multidomain Proapoptotics (BAX, BAK)

The proapoptotic effector proteins BAK and BAX are considered to play redundant functional roles in the initiation of MOMP, as the deletion of either *Bak* or *Bax* alone leads to a minimal level of apoptotic defects (15). However, deletion of both proteins leads to a high incidence of embryonic lethality with surviving mice having a host of developmental and neuronal defects. Not surprisingly, BAX/BAK-deficient mice have a significant increase in both myeloid and lymphoid cells, leading to enlarged primary lymphoid organs and lymphocyte infiltration into peripheral organs (15). Lymphocytes from these animals are resistant to known activators of the intrinsic apoptotic pathway, including cytokine deprivation, etoposide, and irradiation (15). Mice with conditional T cell-specific *Bax/Bak* knockout have abnormal thymocyte development and increased accumulation of double-negative cells in the thymus (16). Thymocytes are resistant to apoptosis following treatment with γ-irradiation and animals develop T cell lymphoma with a median survival of only 10 months (16).

Because BAX and BAK activation is typically considered “the point of no return” in apoptosis induction, therapeutics that can directly activate their oligomerization would be potent initiators of apoptosis. However, there would be a lack of specificity in targeting these proteins directly and off-target effects may limit their clinical use. Direct BAX/BAK activators may find greater efficacy in combination with other, more specific BCL-2 family targeting agents. BH3 mimetics specific for discrete antiapoptotic proteins could potentially lower the apoptotic threshold in a targeted subset of lymphocytes, allowing for lower doses of BAX/BAK activators to exclusively induce apoptosis in these cells. The greatest limitation to this, however, is the inability at the present time to target BH3 mimetics to specific cell populations.

### Multidomain Antiapoptotics (BCL-2, BCL-X_L_, BCL-W, MCL-1, BFL-1)

BCL-2 was the first member of the antiapoptotic subgroup to be extensively characterized. Overexpression of BCL-2 in Eμ*-myc* transgenic mice found that BCL-2 in conjunction with dysregulated c-*myc* promoted immature B cell proliferation and tumorigenesis. However, expression of BCL-2 alone allows for cell survival without any change in proliferation (7). Constitutive BCL-2 expression ultimately leads to increased numbers of pre-B cells, plasma cells, and T cells, all of which demonstrate increased longevity in culture (17, 18). BCL-2 overexpression in these animals leads to autoimmunity similar to that measured in patients with systemic lupus erythematosus (SLE) (17). In contrast, global deletion of *Bcl-2* leads to a significant decrease in the number of double-positive (DP) thymocytes and peripheral (splenic) B and T cells. TUNEL staining of the spleens and thymi of aged *Bcl-2* knockout mice reveals a significant increase in apoptotic cells. Thymocytes lacking BCL-2 are also more susceptible to a wide range of apoptotic stimuli (19).

MCL-1 plays a significant role in immune ontogeny and maintenance. Global deletion of *Mcl-1* is embryonic lethal; therefore, conditional knockout models have been utilized to determine the role of MCL-1 in various immune cell subsets (20). Globally, MCL-1, and not BCL-2, expression is critical for maintaining hematopoietic stem cell survival. Inducible deletion of *Mcl-1* causes rapid bone marrow depletion and mice become moribund within several weeks. These animals rapidly develop severely reduced numbers of hematopoietic stem cells and other bone marrow progenitor populations (21). T cell-specific *Mcl-1* deletion leads to a significant reduction in T lymphocytes, as well as an apparent blockade at the DN2/3 stage of thymocyte development (22). Unlike BCL-2, MCL-1 expression remains constant or is slightly upregulated upon T cell receptor (TCR) stimulation (23, 24). Another distinctive feature of MCL-1 is that it plays a key role in the maintenance of immunosuppressive regulatory T cells (Tregs). Mice with Treg-specific MCL-1 deletion experience weight loss, inflammation, and death due to global autoimmunity within 4–8 weeks (25). Additionally, B cell-specific MCL-1 deletion leads to impaired B cell development beginning at the pro-B cell stage (22). MCL-1 is also essential for the formation of germinal centers and the survival of plasma cells (26, 27).

The remaining antiapoptotic proteins have not been as well characterized in immune system homeostasis. Loss of BCL-X_L_ has a minimal effect on overall T cell survival. While mice lacking BCL-X_L_ have reduced numbers of DP thymocytes, they have normal peripheral lymphocyte numbers indicating that BCL-X_L_ alone is not critical for lymphocyte homeostasis (28). Deletion of *Bfl-1* causes a decrease in DP thymocytes and an increase in double-negative (DN) and CD8^+^ single-positive thymocytes. However, BFL-1-deficient lymphocytes have no significant increase in resistance to apoptotic stimuli (29). Deletion of *Bfl-1* in the myeloid lineage causes a decrease in the granulocyte population and causes increased levels of spontaneous apoptosis in both granulocytes and neutrophils in culture (29, 30). BFL-1 has also been shown to be elevated in several human malignancies, including B cell chronic lymphocytic leukemia (CLL) and familial SLE (31, 32).

**Figure 2:**
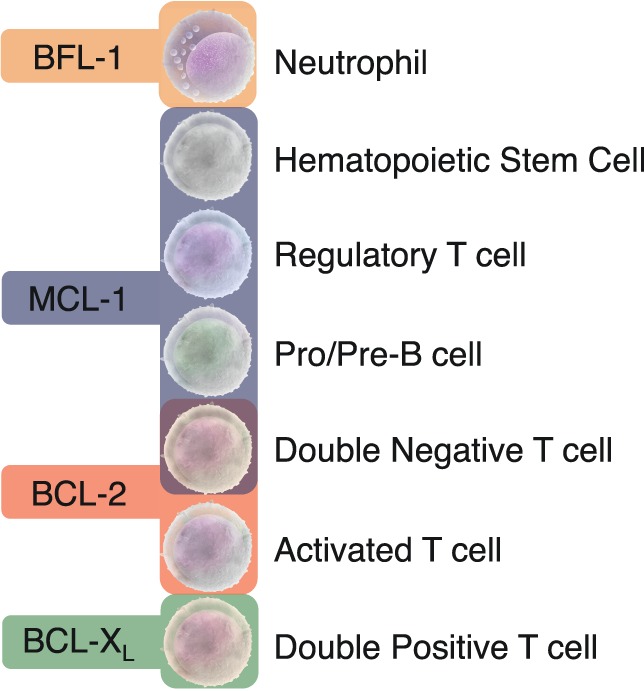
**Distinct dependencies of antiapoptotic BCL-2 proteins exist in specific immune cell subsets**. Animal models with global or conditional deletion of single antiapoptotic proteins have demonstrated that these proteins are essential for unique subsets of immune cells. Most studies have focused on the importance of the BCL-2 family in the lymphoid lineage in both developing and mature B and T cells. The differential dependencies of immune cells on unique antiapoptotic proteins may allow for the targeted drugging of specific immune cell subsets.

Based on the differential reliance on specific antiapoptotic BCL-2 proteins in immune control, it may be feasible to target exclusive subsets of lymphocytes with agents having specificity to the BH3-binding domains of these proteins (Figure 2). Careful consideration will be needed in balancing BH3 mimetic dosing if given together with other chemotherapeutic agents that may lower the therapeutic threshold of immune cells or even alter their antiapoptotic dependency during treatment. The long- and short-term effects of antiapoptotic protein targeting of the immune system is unknown and how other proteins in this subclass may compensate is unclear. Rapid upregulation of non-targeted antiapoptotic proteins has been found to occur in lymphocytes when treated with BH3 mimetics (33). How this will impact immune effects clinically has yet to be determined.

### BH3-Only Proteins (BIM, BID, PUMA, BAD, NOXA, BMF)

Most data regarding the role of BH3-only proteins has involved studying the direct activator proteins BIM, BID, and PUMA though genetic deletion in murine models. BIM is considered the master regulator of immune cell homeostasis. *Bim* deletion causes increased numbers of lymphoid and myeloid cells, defects in thymocyte development, and global lymphocyte resistance to multiple apoptotic stimuli (34, 35). BIM is essential for negative selection of immature T cells in the thymus, and mice lacking BIM have shorter lifespans due to the development of fatal autoimmunity (34, 36). BIM also plays an important role in regulating the survival of CD4^+^ T cells in the periphery. Decreased BIM expression with age leads to longer-lived CD4^+^ T cells that are prone to functional defects and become increasingly unable to properly respond to pathogens (37). Deletion of additional BH3-only proteins exacerbates the immune dysfunction seen in *Bim* knockout animals. Combined deletion of *Bim* and *Puma* causes increased resistance to a wide range of apoptotic stimuli, and a subset of these mice develop spontaneous follicular B cell lymphoma (38). Triple knockout of *Bim, Puma*, and *Bid* leads to increased lymphocytosis and profound, yet not complete, apoptotic resistance (39). The prevalence of hematological malignancies has not yet been characterized in these triple knockout mice (39).

BID is a unique BH3-only protein because its structure is more similar to the multidomain antiapoptotics and it provides functional cross talk between the intrinsic and extrinsic apoptotic pathways. BID is cleaved to its functional form, tBID, by activated caspase-8, downstream of plasma membrane death receptor activation (40). Young mice deficient in BID have no major hematological defects, whereas aged mice develop neutrophilia and many succumb to a hematopoietic malignancy resembling human chronic myelomonocytic leukemia (CMML) (41). BID may also play a key role in survival of Langerhans cells, a unique subset of dendritic cells, as BID-deficient Langerhans cells are more resistant to CD4^+^ T cell-mediated apoptosis (42).

PUMA is regulated by the tumor suppressor p53 and has been implicated as a mediator of apoptosis in several subsets of immune cells (43, 44). PUMA cooperates with BIM to regulate activated T cell contraction following an immune response. Mice lacking PUMA accumulate activated CD8^+^ T cells in their spleen following herpes simplex virus (HSV-1) infection, and these cells are more resistant to cytokine deprivation in culture (45). PUMA is also upregulated in activated B cells and *Puma* deletion leads to apoptotic resistance and B cell accumulation in addition to increased levels of memory B cells following antigen stimulation (46). In the myeloid compartment, PUMA deficiency has been shown to impair the regulation of neutrophil contraction following an immune response, thus compromising the ability to properly respond to bacterial infections, which can lead to lethal sepsis (47).

Similar to PUMA, the indirect activator NOXA is regulated by p53 (48). *Noxa* knockout mice have no aberrations in thymocyte development; however, NOXA may play a minor role in regulating the formation and maintenance of effector memory T cells during and following an immune response (49, 50). NOXA has also been shown to play a key role in B cell activation and efficient generation of high-affinity antibody clones. Loss of *Noxa* leads to an accumulation of low affinity B cells due to dysregulated apoptosis of these cells during immune response initiation (51).

Normal B and T cell development is maintained in cells lacking BAD and lymphocytes from BAD-deficient animals retain normal sensitivity to apoptotic stimuli (52). Currently, BAD appears most important in B cell ontogeny and maturation. Although incompletely understood, *Bad* knockout mice have reduced IgG production after lipopolysaccharide (LPS) stimulation and aged mice develop diffuse large B cell lymphoma (DLBCL) that is increasingly penetrant following ionizing radiation (52).

Although not as extensively studied as other BH3-only proteins, *Bmf* knockout mice maintain normal overall T cell counts and have no known abnormalities in thymocyte development but do experience B cell hyperplasia. BMF-deficient T- and pre-B cells are resistant to apoptosis in response to glucocorticoids or HDAC inhibition (53). Mice lacking BMF also develop thymic lymphomas following exposure to γ-irradiation (53).

Extensive characterization of the BH3-only proteins in the immune system has revealed both overlapping and non-redundant roles for many of these proteins in specific immune cell subsets. Major focus has been placed on designing therapeutics that mimic the binding of these proteins to multidomain apoptotic effectors. As development of these compounds increases for use as single agent or combination anti-cancer therapeutics, it will be essential to continue defining the exact roles of the BH3-only proteins in the ontogeny and maintenance of clinically relevant anti-viral, anti-bacterial, and antitumor immune responses. How these proteins are displaced following treatment and sequestered by expressed antiapoptotics in immune cell subtypes is of particular interest. Harnessing their immune-based “off-target” effects could allow for powerful modulation of the immune system alone or in concert with other emerging immune-based therapies.

## BH3 Mimetics as Anticancer Therapeutics

The BCL-2 family of proteins are heavily implicated in tumorigenesis and targeting their interactions shows great promise in overcoming apoptotic resistance in a vast array of malignancies (54, 55). Given the importance of BH3-only proteins in regulating the cross talk between the anti- and proapoptotic multidomain BCL-2 proteins, most drug development has centered on recapitulating their mechanism(s) of action in cells. These so called “BH3 mimetics” encompass an array of natural products, small molecules, and peptide therapeutics that mimic the BH3-domain-directed binding of BCL-2 proteins to either lower the apoptotic threshold or directly initiate the intrinsic apoptotic cascade. Most work has focused on the role of these compounds as anticancer therapeutics as described below. Less well understood is how these compounds can be used to modulate normal immune responses. It is important to consider that cancer cells are “primed to die” due to their extreme dependence on thwarting apoptosis given their genetic and metabolic abnormalities. In contrast, although susceptible to these compounds, normal lymphocytes may have different sensitivities to these drugs as compared to malignant cells. It is critical that these potential differences are further elucidated in order to generate the most effective strategies for immune modulation.

### Compounds in Clinical Trials

#### Antisense Oligonucleotides

##### Oblimersen Sodium (Genasense)

Over 25 years ago, it was demonstrated that an antisense oligonucleotide targeting BCL-2 could abrogate *in vivo* tumor growth (56). These studies led to the development of the optimized antisense oligonucleotide oblimersen sodium (G3139; genasense; augmerosen), which demonstrated *in vitro* and *in vivo* efficacy against multiple hematological malignancies, including non-Hodgkin’s lymphoma (NHL), EBV-associated lymphoproliferative disorders, and Philadelphia chromosome-positive leukemia (57–59). After showing promise in multiple preclinical models, oblimersen was tested in clinical trials for both hematological malignancies and solid tumors. Phase I and II clinical trials were encouraging against a wide range of cancer types, including hormone-refractory prostate cancer, CLL, and NHL (60–62). Unfortunately, despite initial promise, several phase III studies have found that oblimersen does not improve the response rate seen with the current standards of care (63, 64). Oblimersen has not been FDA approved and subsequent attention has turned to the development of small molecules and peptide therapeutics that directly disrupt intracellular BCL-2 family protein:protein interactions.

#### Small Molecules

##### ABT-737 and ABT-263 (Navitoclax)

The first small molecule to effectively mimic the interaction between antiapoptotics and BH3-only proteins was the BAD-derived BH3-only mimetic ABT-737 (65). Discovered using a nuclear magnetic resonance (NMR)-based screening method, this small molecule has high affinity for the BH3-binding pocket of BCL-2, BCL-X_L_, and BCL-W (65). ABT-737 has been shown to disrupt BAX/BCL-2 complexes, leading to the release of cytochrome *c* and the initiation of the caspase cascade (66, 67). BAX/BAK double knockout cells administered ABT-737 experience no significant decrease in viability, indicating that ABT-737 functions through on-target binding to antiapoptotic proteins to induce the intrinsic mitochondrial apoptotic pathway (67). ABT-737 has potent *in vitro* activity against a wide range of hematological malignancies, including acute myeloid leukemia (AML), multiple myeloma (MM), and acute lymphoblastic leukemia (ALL) (67–69). Subsequent studies in xenograft models of adult and pediatric hematological diseases have confirmed the on-target potency of ABT-737 against malignant cells (70–72). The oral analog of ABT-737, ABT-263 (navitoclax), has been tested in clinical trials for both hematological and solid tumors (73). However, patients experienced dose-limiting thrombocytopenia due to the dependency of platelets on BCL-X_L_ and BAK (74, 75). Another important caveat is that many cancers are refractory or become resistant to ABT-737 or ABT-263 due to upregulation of antiapoptotic proteins (e.g., MCL-1, BFL-1) that lack specificity to either compound (67, 76, 77). It will therefore be imperative to consider which antiapoptotic proteins target cells express and understand their real-time compensatory capacity for apoptotic resistance before treatment with BH3 mimetics having limited antiapoptotic protein specificity.

##### ABT-199 (Venetoclax)

To overcome the thrombocytopenia caused by ABT-263, a new BCL-2-specific BH3 mimetic was derived based on the X-ray crystal structure of BCL-2 and ABT-263 (78). ABT-199 binds BCL-2 with subnanomolar affinity (78). ABT-199 has demonstrated effective *in vitro* and *in vivo* cell killing in a range of cancers, including chronic myelogenous leukemia (CML), AML, and T-cell ALL (79–81). Based on its efficacy in clinical studies, ABT-199 has recently gained FDA approval for the treatment of refractory 17-p-deleted CLL, making it the first clinically approved small molecule targeting intracellular protein:protein interactions (82). Interestingly, there are reports describing ABT-199 inducing cell death in normal immune cell subsets in addition to its desired anticancer activity as was found in the case of normal mature B cells isolated from patients with CLL (83).

##### GX15-070 (Obatoclax)

The first pan inhibitor of the antiapoptotic BCL-2 proteins was GX15-070 (84). GX15-070 binds all antiapoptotics with nanomolar to low micromolar affinity and, importantly, is able to overcome ABT-737 apoptotic resistance in cells with high MCL-1 expression (85, 86). GX15-070 has efficacy against a range of solid tumors and hematological malignancies (86–88). GX15-070 was well tolerated in phase I clinical trials for patients with CLL, refractory leukemia, and myelodysplasia (89, 90). Unfortunately, in phase II and phase I/II clinical trials, GX15-070 did not improve outcomes in patients with myelofibrosis, mantle cell lymphoma, or AML (91–93). On target specificity has been questioned regarding the mechanism of action of GX15-070, as BAX/BAK double knockout cells die when treated with this compound (87). In fact, multiple modes of cell death have been measured in response to GX15-070 treatment. Canonical features of apoptosis, necrosis, and autophagy are seen in infant ALL (*MLL-*rearranged ALL) patient samples following treatment, indicating activation of related but not overlapping cell death mechanisms (94).

#### Natural Products

##### Gossypol Family

Gossypol is a natural phenolic pigment isolated from cottonseed. Its negative enantiomer R-(−)-gossypol, or AT-101, binds BCL-2, BCL-X_L_, BCL-W, and MCL-1 and may have efficacy as an anticancer therapeutic (95, 96). Like GX15-070, gossypol may not induce cell death exclusively *via* the intrinsic apoptotic pathway because BAX/BAK double knockout cells die following treatment (97). Despite potential off-target effects, gossypol has shown efficacy against several hematological malignancies, including DLBCL, MM, and CLL (98–100). Gossypol has entered several clinical trials, including a phase II trial for small cell lung cancer; however, the results have not been promising (101). Subsequent studies have focused on the development of small molecules derived from gossypol in order to improve its potency and potential clinical efficacy (102, 103). Interestingly, it has been demonstrated that gossypol induces apoptosis in polymorphonuclear leukocytes and monocytes isolated from healthy donors, suggesting a future potential as an immune modulator (104).

#### Preclinical Compounds

The promising clinical results of the aforementioned BCL-2 modulators have driven the discovery of a diverse range of small molecules and peptide therapeutics currently in clinical and preclinical development (Table 1). New small molecules have been designed as specific inhibitors of single or multiple antiapoptotic proteins. Single protein inhibitors will be useful for targeting malignant cells that are highly dependent on one antiapoptotic protein with theoretically minimal off-target effects. However, malignant cells not initially killed have been shown to rapidly upregulate antiapoptotic proteins lying outside of the primary compound’s binding profile. Pan apoptotic inhibitors may have increased potency but run the risk of greater off-target cell killing. In the context of immune modulation, targeting single antiapoptotics may be more desirable in manipulating the immune response over time and may allow for greater therapeutic dissection of specific myeloid, T, and B cell subpopulations.

**Table 1:** **Clinical and preclinical BH3 mimetics**.

Class	Compound name	Known target(s)	Reference
**Clinical**
Antisense oligodeoxynucleotide	Oblimersen sodium	BCL-2	(57–59)
Small molecule	ABT-737/263	BCL-2, BCL-X_L_, BCL-W	(65, 67, 73)
	ABT-199	BCL-2	(78, 82)
	Obatoclax/GX15-070	BCL-2, BCL-X_L_, BCL-W, MCL-1, BFL-1	(84, 86)
**Preclinical**
Natural product	Gossypol	BCL-2, BCL-X_L_, BCL-W, MCL-1, BFL-1	(95, 96)
Small molecule	WEHI-539	BCL-X_L_	(105)
	BXI-61/72	BCL-X_L_	(106)
	A-1155463	BCL-X_L_	(107)
	TW-37	MCL-1	(108)
	MIM-1	MCL-1	(109)
	A-1210477	MCL-1	(110)
	Maritoclax	MCL-1	(111)
	Compound 21	MCL-1	(112)
	2-Indole-acylsulfonamides	MCL-1	(113)
	Agossypol	BCL-2, BCL-X_L_, BCL-W, MCL-1	(102)
	Apogossypolone (ApoG2)	BCL-2, BCL-X_L_, MCL-1	(114)
	BI97D6	BCL-2, BCL-X_L_, MCL-1, BFL-1	(115)
	Sabutoclax/BI-97C1	BCL-2, BCL-X_L_, MCL-1, BFL-1	(116)
	BM-1197	BCL-2, BCL-X_L_	(117)
	S1	BCL-2, MCL-1	(118)
	BH3-M6	BCL-2, BCL-X_L_, MCL-1	(119)
	JY-1-106	BCL-X_L_, MCL-1	(120)
	BAM-7	BAX	(121)
Peptide therapeutic	072RB	BCL-X_L_	(122)
	XXA1	BCL-X_L_	(123)
	Biphenyl-cross-linked NOXA peptide	MCL-1	(124)
	MCL-1 SAHB*_D_*	MCL-1	(125)
	BIM SAHB*_A_*-3	BFL-1	(126)
	BIM SAHB*_A_*(146–166)	BCL-2, BCL-X_L_, BCL-W, MCL-1, BFL-1, BAX	(127)
	PUMA SAHB*_A1_*	BCL-2, MCL-1, BAX	(128)

In addition to small molecules, there is increasing interest in the use of peptide-based BCL-2 therapeutics that mimic the binding interface of specific BH3-only:antiapoptotic complexes. Isolated native BH3 helices are not attractive pharmaceutical compounds as they do not typically maintain their helical structure, are quickly degraded, and lack cellular penetrability (129). Therefore, it is necessary to chemically modify these peptides in order to maintain their secondary structure, binding affinity, and protease resistance as has been done through chemical hydrocarbon stapling, chemical cross-linking, or peptide amphiphile/micelle incorporation (124, 129). While BCL-2 peptides have yet to reach clinical testing, the ability to target larger surface areas of protein:protein interactions is a promising and highly specific targeting strategy.

## BH3 Mimetics as Immune Modulators

Most studies to date measuring the immune effects from BCL-2 modulation have tested ABT-737 in the context of autoimmunity or transplant tolerance and emphasize the compound’s effects on lymphocytes (T and B cells). One of the first studies to examine the potential for BH3 mimetics to target the immune system found that treatment with ABT-737 induces apoptosis in lymphocytes and reduces the severity of disease in several murine models of autoimmunity (130). Specifically, treatment with ABT-737 significantly reduces paw swelling in mice with collagen-induced arthritis and improves overall survival and renal function in mice with a SLE-like syndrome (130, 131). ABT-737 also suppresses immune responses to immunization against keyhole limpet hemocyanin (KLH), as T cells isolated and restimulated from ABT-737-treated mice have a significant reduction in proliferation upon re-exposure to KLH (130). Subsequent studies on the effects of ABT-737 on normal hematopoietic compartments have shown that treatment causes a significant reduction in CD4^+^ and CD8^+^ T cells, B cells, and some subsets of dendritic cells, thus perhaps diminishing proper antigen presentation and T and B cell expansion (132). In fact, treatment with ABT-737 leads to prolonged pancreatic islet allograft survival in a murine model of spontaneous diabetes, and animals are able to maintain normal long-term control of blood glucose levels compared to vehicle-treated controls (132). While ABT-737 treatment alone is able to suppress allogeneic T cell responses *in vitro*, treatment with ABT-737 *in vivo* acts synergistically with cyclosporine A to reduce skin graft rejection in a MHC mismatched transplant model (133). Interestingly, ABT-737 has also been shown to preferentially induce apoptosis in conventional T cells (Tcons) leading to a relative enrichment of immunosuppressive Tregs. This enrichment was found to slow progression in a murine model of graft-versus-host disease (GVHD) and improve overall survival following hematopoietic stem cell transplantation (134). In addition to potential benefits in transplantation and autoimmunity, ABT-737 may be useful in the mediation of inflammatory diseases. ABT-737 induces apoptosis in T lymphocytes and lamina propria mononuclear cells in a BIM-dependent manner, which is able to reduce levels of inflammation in spontaneous (IL-10^−/−^) and acute models of colitis (135).

In addition to lymphocytes, ABT-737 also has the potential to affect mature cells of other hematopoietic lineages. Mast cells are specialized myeloid cells that sense pathogens and initiate inflammatory responses. Their dysregulation can lead to aberrant inflammation and allergic reactions (136). Different mast cell populations are sensitive to ABT-737 at varying dosages (137). As expected, human and murine mast cell resistance to ABT-737 correlates with decreased BCL-2 and increased MCL-1 expression (137). Interestingly, *in vivo* analysis indicates that while mast cells were highly sensitive to ABT-737, T cells isolated from the peritoneum of treated mice are unaffected by ABT-737. Other studies have demonstrated a marked reduction in T cells isolated from lymphoid organs and peripheral blood following ABT-737 administration, indicating that immune cell localization and tissue environment may be a critical factor in determining sensitivity to BH3 mimetics (132, 133). These parameters should be carefully considered when testing the *in vivo* efficacy of these compounds prior to clinical translation for immune control.

Because of apparent Treg dependency on MCL-1, recent work has used GX15-070 to differentially target Tregs over Tcons. T cell sensitivity to GX15-070 appears to vary *in vitro* depending on a T cell’s activation status. Mature human T cells that have undergone prolonged activation are more resistant to GX15-070 compared to lymphocytes in the early stages of activation (138). This sensitivity profile extends to peripheral blood mononuclear cells (PBMCs) isolated from patients with ovarian cancer. *In vitro* treatment leads to significantly increased CD8^+^:Treg and CD4^+^:Treg ratios, indicating that GX15-070 preferentially induces apoptosis in the Treg subpopulation (138). Depletion of Tregs while preserving Tcons is a promising therapeutic strategy for amplifying the antitumor immune response. These results support the finding that *in vivo* treatment with GX15-070 following vaccination leads to decreased lung metastases in a murine model of lung adenocarcinoma (139).

These studies emphasize the promising immunomodulatory potential of BH3 mimetics. More extensive testing using a wider range of small molecules and peptide therapeutics with varying antiapoptotic specificities will provide a fuller understanding of the mechanisms responsible for clinically effective immune control.

## Surmising Immune Effects from Clinical Trials with BH3 Mimetics

Although most published clinical reports using BCL-2 therapeutics have concentrated on their antitumor effects, BH3 mimetic-induced manipulation of immune surveillance and activation could have profound ramifications for people suffering from a myriad of immunologically mediated conditions. Understanding the specific effects on leukocyte subsets of patients treated with these compounds will be paramount for their effective clinical translation. Beyond the results from testing these compounds *in vitro* and in preclinical animal models, most of what we currently know about the effects of these compounds on the human immune system must be extrapolated from oncology-based clinical trials.

Scrutinizing these studies indicate that targeted effects on the immune system include significant lymphopenias and neutropenias. Lymphopenia is a desirable effect in many cases, especially when treating hematologic malignancies like CLL. Patients with relapsed or refractory CLL treated with ABT-199 all had a >50% reduction in their absolute lymphocyte counts (ALC) and the majority had a 100% reduction. Neutropenia (grades 3 or 4) was reported in 35% of the patients with 6% of the patients experiencing serious infections and 2.5% developing autoimmune neutropenia (140). While ABT-263 induces on-target thrombocytopenia, it also elicits neutropenia 28% of the time with many patients developing subsequent infectious sequelae (141, 142). Similar findings have been observed in patients treated with other BH3 mimetics at antitumor dosing, including AT-101 and GX15-070 (89, 143).

Phase I clinical trials using the BCL-2 mRNA-targeting oligonucleotide oblimersen found that treatment with this compound led to global myelosuppression including neutropenia and lymphopenia (144, 145). However, a caveat to these and other trials is that these studies included concurrent cytotoxic chemotherapy making it difficult to specifically analyze the effects of BCL-2 targeting alone on the immune system. Of note, oblimersen has been tested in pediatric patients with neuroblastoma making it the first compound targeting BCL-2 in children (61, 144, 145). Dose escalation phase I studies evaluating the safety of the BCL-2 deoxyribonucleic acid inhibitor (DNAi) PNT2258 in patients with advanced solid tumors found rapid (within hours) decrease in lymphocytes with most patients having a >50% reduction. This phenomenon was dose dependent and used as a surrogate for efficacy (146).

Outside of the oncologic arena, BCL-2 family modulation using ABT-199 has been tested in patients with SLE. Most data remains preliminary, such as those presented at a recent American College of Rheumatology Annual Meeting (2015) demonstrating a dose-dependent reduction in total lymphocytes and B cells in particular. Neutropenia also occurred but was less consistent and correlated to different dosing thresholds (www.clinicaltrials.gov identifier NCT01686555) (147).

Clinical trials using direct BCL-2 modulation are on the rise. Searching www.clinicaltrials.gov as of August, 2016 found a number of active trials testing ABT-199 (15 trials), ABT-263 (4 trials), and gossypol/AT-101 (3 trials). Searching “oblimersen”/“genasense” or “obatoclax”/“GX15-070” revealed no entries. However, all of these active trials are testing these drugs as anticancer agents and none are directly investigating their effects on immune function or autoimmune disease. As the number of clinically available BH3 mimetics continues to grow and we further expand our knowledge regarding how these compounds affect specific immune cell subsets, we can expect a robust increase in the clinical trials evaluating BH3 mimetics in the context of immune modulation.

## Future Prospects

**Figure 3:**
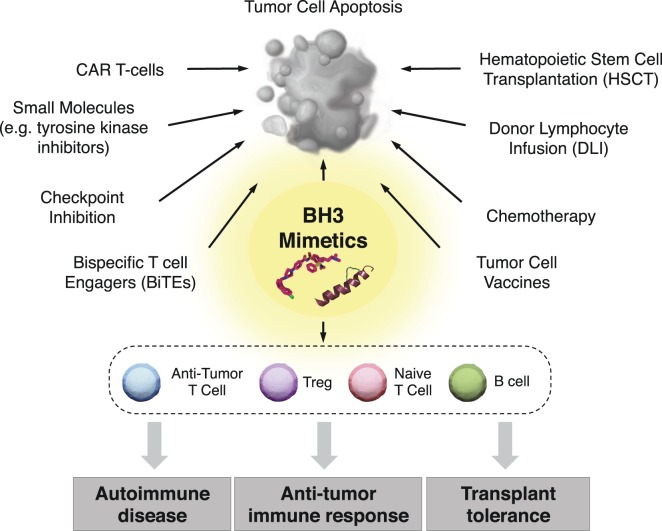
**Clinical implementation of BH3 mimetics for immune modulation**. In addition to their use as anticancer therapeutics, BH3 mimetics have promise for targeting specific immune cells subsets, which may provide therapeutic benefit in the context of antitumor immune responses, transplantation tolerance, and autoimmune diseases. Additionally, BH3 mimetics may be combined with both classical and cutting edge chemo- and immunotherapeutics to improve the standards of care in patients with a wide range of hematological malignancies.

BH3 mimetics represent a unique arsenal of compounds with a wide range of potential therapeutic interventions beyond that of directly inducing malignant cell death. Determining the effects of long-term selective pressure on the immune system is gaining significance as patients are increasingly treated with this class of drugs. BH3 mimetics could potentially be used alone or in combination with classic immunotherapies such as stem cell transplantation and donor lymphocyte infusions, or with other more advanced therapies such as checkpoint inhibition, cancer vaccines, antibody-based therapies, chimeric antigen receptor (CAR) T-cells, and bispecific T cell engagers (BiTEs) antibodies (148) (Figure 3).

However, there are clinical considerations and unanswered mechanistic questions that should be addressed as BH3 mimetics become effective clinical immunomodulators. First, while several studies with ABT-737 and GX15-070 have been performed to determine how they affect immune responses, there is an ever-expanding range of BH3 mimetics with diverse BCL-2 protein specificities. How these agents affect immune cell subsets as single agents or in combination is unclear. Second, resistance to a single BH3 mimetic is a reoccurring problem in cancer cells due to the ability of antiapoptotic proteins to rapidly compensate for functional loss of another (33). Determining if this occurs in non-malignant immune cells, the kinetics of change, and the functional significance of BCL-2 repertoire shifts in immune cell subsets in response to BH3 mimetic treatments will be critical in order to determine if resistance to these therapies will develop over time. BH3 peptide profiling is a powerful method that allows for the determination of the antiapoptotic proteins upon which a cell is dependent (149, 150). However, whether performed on isolated mitochondria or semi-intact cells, this method alone does not conclusively determine BH3 dependency or sequestration in real time. Use of BH3 profiling in conjunction with other described methods such as qRT-PCR, quantitative fluorescence cytometry, and BIM:antiapoptotic dissociation analysis should allow for a more complete assessment of apoptotic dependency throughout treatment (151, 152).

Other treatment-related questions include timing, dosing schedule, and combination therapy. The ideal timing of BH3 treatment in conjunction with other immunotherapies will need to be delineated. Single dosing versus metronomic dosing may have a major impact on the effectiveness of the treatment, severity of off-target effects, and emergence of resistance. Assessing initial dependency and following this long term under constant pressure from mimetic treatment may allow for increasingly thoughtful decisions regarding the best way to implement these compounds in the clinic. In fact, it may be possible to “prime” cells to die by treating with BH3 mimetics at lower doses, thus causing upregulation of potential resistance factors (for example, treating with ABT-737 may cause MCL-1 or BFL-1 upregulation). Subsequent treatment with a complementary compound targeting these resistance factors (an MCL-1 or BFL-1-specific compound in this example) may lead to even more potent cell killing or sequential immune cell modulation. The potential efficacies of these strategies remain largely unexplored.

Finally, combining BH3 mimetics with conventional chemotherapy, DNA/histone-modifying drugs (e.g., HDAC inhibitors, methyltransferase inhibitors), and/or small molecules (e.g., tyrosine kinase inhibitors) may offer even further differential immune effects when used at lower doses, thus decreasing overall therapeutic toxicities. Such combination therapy may also prevent evolution of BCL-2 resistance within the immune system as well as within the primary malignancy. Combining BH3 mimetics with other immune-directed therapies such as rituximab, checkpoint inhibitors, ibrutinib, calcineurin, and mTOR inhibitors may allow for even greater immunological fine-tuning, as the apoptotic repertoires and BH3 mimetic sensitivities in specific immune subsets are likely to change in response to these drugs. Overall, as greater preclinical and clinical understanding of the effects of BH3 mimetics on the immune system expands, we can expect to usher in new and exciting immunomodulatory capacities of this drug class either when used alone or in conjunction with other treatments.

## Author Contributions

LL, MN, AH, and JL: all the authors listed have made substantial, direct, and intellectual contribution to the work and approved it for publication. LL and JL drafted the final work and revised it critically for content.

## Conflict of Interest Statement

The authors declare that the research was conducted in the absence of any commercial or financial relationships that could be construed as a potential conflict of interest.
